# Joint effects of leukocyte-albumin ratio and hypertension are associated with multidimensional cognitive decline and Alzheimer’s disease risk in older adults: evidence from NHANES and a clinical AD validation cohort

**DOI:** 10.3389/fnagi.2026.1826100

**Published:** 2026-08-12

**Authors:** Pan Gao, Yiming Chu, Mingjun Geng, Nannan Tian, Diandian Chen, Yingli Si, Heyang Liu, Peichang Wang, Jingrong Cao

**Affiliations:** 1 Department of Clinical Laboratory, Xuanwu Hospital, Capital Medical University, Beijing, China; 2 Department of Clinical Laboratory, Xiongan Xuanwu Hospital, Xiongan New Area, Hebei, China

**Keywords:** Alzheimer’s disease, biomarker, cognitive impairment, hypertension, joint effects, leukocyte-albumin ratio, NHANES cohort, validation cohort

## Abstract

**Background:**

Alzheimer’s disease (AD) pathogenesis involves complex interactions between neuroinflammation and vascular dysfunction. While leukocyte-albumin ratio (LAR) and hypertension are independently linked to cognitive decline, their joint associations with multidimensional cognitive impairment and AD risk remain understudied.

**Methods:**

We analyzed two cohorts: 1,300 adults (≥ 60 years) from NHANES 2011–2014, and a clinical cohort (50 AD patients + 125 age-/gender-matched controls). LAR was calculated, hypertension was defined as per JNC7 criteria, and cognitive function was assessed via AD-sensitive tests standardized to *z*-scores. Restricted cubic spline (RCS) regression, multivariable linear regression, subgroup analysis, and Cox regression were used to examine LAR, hypertension, and their combined associations with cognitive outcomes.

**Results:**

In the NHANES cohort, LAR was significantly correlated with DSST performance; participants with co-occurring high LAR + hypertension had lower scores across three cognitive domains, with age- (65–74 years) and sex-specific patterns and a 2.17-fold higher all-cause mortality risk. In the validation cohort, AD patients had higher LAR, with the highest LAR quartile linked to a 3.92-fold increased AD risk (*p* = 0.015).

**Conclusion:**

Our findings support an association between LAR, hypertension, and cognitive decline. Elevated LAR in conjunction with hypertension is associated with poorer cognitive performance across multiple domains in older adults, as observed in both the NHANES and a clinical AD cohort. As a low-cost, easily measurable biomarker, LAR may hold promise for early AD risk stratification in primary care, highlighting a potential target for combined anti-inflammatory and antihypertensive interventions. However, its modest discriminative ability (AUC = 0.61) indicates that LAR should not be used as a standalone diagnostic tool. Prospective longitudinal studies are warranted to validate these associations.

## Introduction

Alzheimer’s disease (AD) is the most common form of dementia, affecting over 55 million people globally and accounting for 60 ~ 80% of all dementia cases (Livingston et al., 2020). Although AD is characterized by extracellular beta-amyloid (Aβ) plaques and intraneuronal tau tangles, its pathogenesis increasingly emphasizes early, modifiable mechanisms, including neuroinflammation and vascular dysfunction (González et al., 2023; Jin and Bhaskar, 2025). Hypertension, a leading vascular risk factor, disrupts blood–brain barrier (BBB) integrity, promoting neuroinflammatory infiltration and neuronal damage (Mayer and Fischer, 2025). Systemic inflammation (elevated leukocytes) and nutritional imbalance (reduced albumin) are independently linked to cognitive decline and AD risk (Jacobs et al., 2024; Boada et al., 2020), but their synergistic effects remain unexplored.

The leukocyte-albumin ratio (LAR) is a novel composite inflammatory-nutritional index derived from routine laboratory measurements. By integrating systemic inflammation (leukocyte count) and nutritional status (albumin level) into a single metric, LAR offers a more comprehensive assessment than individual markers (Lessomo et al., 2023). LAR has been reported as an independent predictor of major adverse cardiovascular events (MACEs), including myocardial infarction, stroke, and heart failure, with incremental prognostic value beyond traditional risk factors (Maldonado-Díaz et al., 2024). The limited existing research on LAR in cognitive impairment includes three small cross-sectional studies that showed elevated LAR in AD patients compared to controls. While these three small cross-sectional studies have reported elevated LAR in AD patients, prior research has focused primarily on the isolated effects of LAR or hypertension. Their joint association with multidimensional cognitive decline and AD risk remains unknown. This knowledge gap limits the development of targeted risk stratification strategies for early AD, where modifiable factors have the greatest impact (Garcia-Bustos et al., 2025).

Here, we hypothesize that elevated LAR and hypertension may jointly associate with cognitive decline in older adults, particularly in domains vulnerable to vascular and inflammatory damage. We tested this hypothesis using a nationally representative NHANES cohort and a clinical validation cohort of AD patients, with the following aims: (1) to characterize the dose–response relationship between LAR and cognitive *z*-scores and determine whether this association was linear or non-linear using restricted cubic spline analysis; (2) to assess the association of combined LAR and hypertension status with cognitive *z*-scores using multivariable linear regression models; (3) to perform pairwise comparisons among the study groups to further evaluate intergroup differences; and (4) to investigate differences in survival outcomes across the four groups defined by LAR and hypertension status using Kaplan–Meier survival analysis and Cox proportional hazards models.

## Materials and methods

### Study population and cohort design

**Figure 1:**
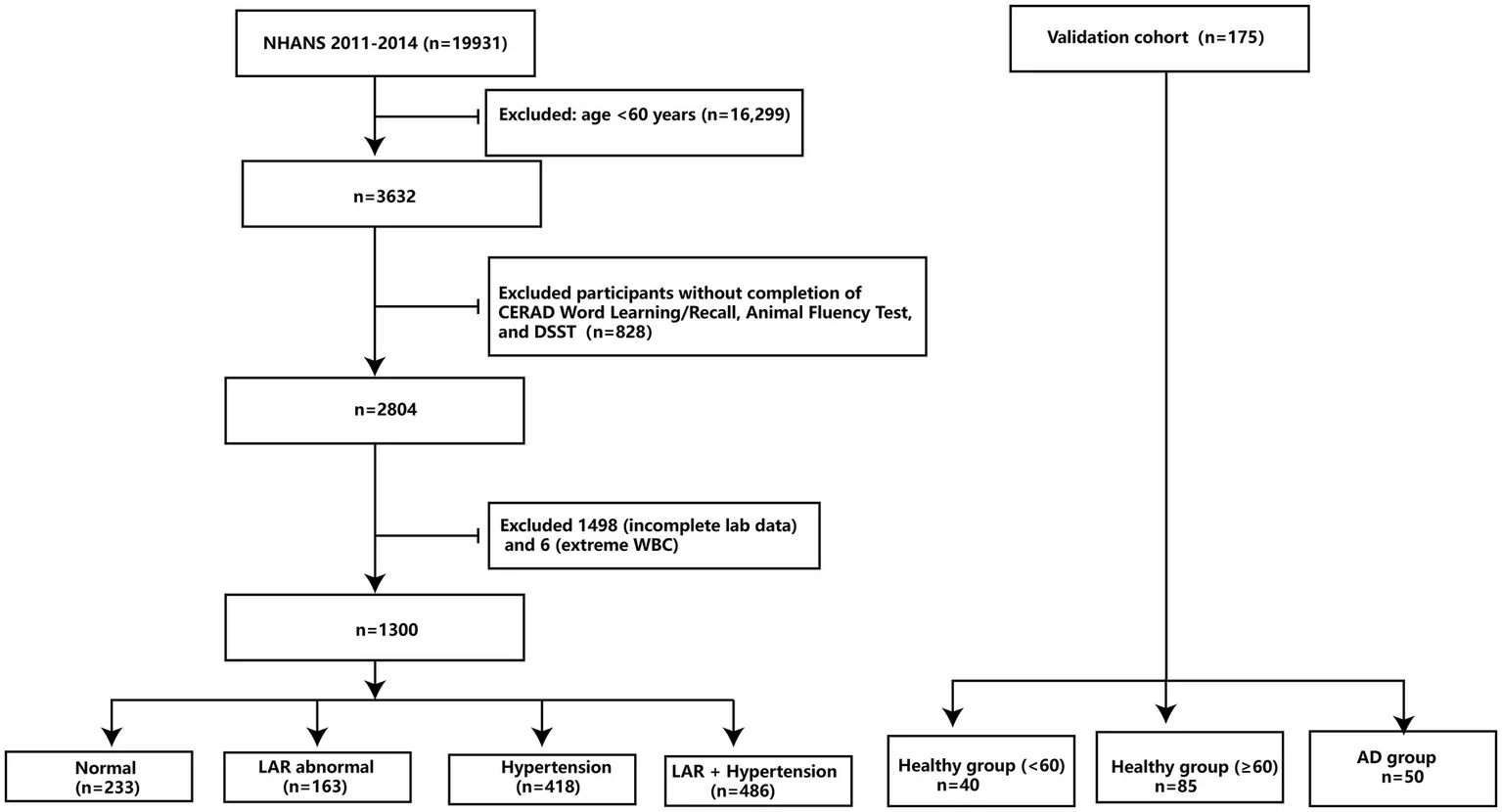
Study population inclusion and exclusion flowchart.

We analyzed data from two independent cohorts: (1) National Health and Nutrition Examination Survey (NHANES) Cohort: Eligible participants were aged ≥ 60 years with complete cognitive assessments and laboratory data from the NHANES 2011–2014, a nationally representative cross-sectional study of the US non-institutionalized population. NHANES protocols were approved by the National Center for Health Statistics (NCHS) Research Ethics Review Board, and informed consent was obtained from all participants.^1^ The final analytic sample included 1,300 participants. (2) Validation cohort: The clinical validation cohort included 50 patients with AD diagnosed according to the National Institute of Neurological and Communicative Disorders and Stroke and the Alzheimer’s Disease and Related Disorders Association (NINCDS-ADRDA) criteria (McKhann et al., 2011), and 125 age- and gender-matched cognitively healthy controls recruited from Xuanwu Hospital. Data on physical activity, dietary intake, and detailed hypertension characteristics (duration, grade) were not systematically collected as part of the retrospective study design. The study protocol was approved by the Medical Ethics Committee of Xuanwu Hospital ([2025] 072-001), and written informed consent was obtained from all participants or their legal representatives. The study workflow is presented in Figure 1.

### Sample size and statistical power calculation

*Post-hoc* power analysis using G*Power 3.1. Sample size calculation confirmed 80% statistical power to detect an effect size of 0.5 at *α* = 0.05.

### Definition of key variables

*LAR calculation*: LAR = Leukocyte count (×10^9^/L) /albumin level (g/L). Extreme values (±3 SD from the mean) were excluded to reduce bias. Whole blood counts were measured using the Sysmex XN-1000/9000 hematology analyzer, with coefficient of variation (CV) < 2% for leukocyte counts. Serum albumin was measured using the bromocresol green method on a Hitachi 7600 chemistry analyzer, with inter-assay CV < 1.5%.

*Hypertension diagnosis*: Defined per Seventh Report of the Joint National Committee (JNC7) criteria (Chobanian et al., 2003): systolic blood pressure (SBP) ≥ 140 mmHg, and/or diastolic blood pressure (DBP) ≥ 90 mmHg, or self-reported diagnosis, or antihypertensive medication use.

*Cognitive function assessment* (Jaeger, 2018): (1) Episodic memory: the Rey Auditory Verbal Learning Test (RAVLT) was administered according to standard protocols: Participants heard a list of 10 unrelated nouns, with immediate word recall tested after each of 5 learning trials and delayed recall test 30 min later. The score represents the number of correctly recalled words. (2) Semantic memory and executive function: Participants were given 60 s to name as many animals as possible to test animal fluency. The score is the total number of unique animals named. (3) Executive function/processing speed: The Wechsler Adult Intelligence Scale (WAIS) version of the digit symbol substitution test (DSST) was used, with 133 possible items completed in 120 s. The score is the number of correct substitutions.

*Joint and interaction effect analysis*: To distinguish joint associations from formal multiplicative interactions, two separate analytical approaches were used. First, for the joint association analysis, participants were classified into four groups according to dichotomized LAR status (lower quartiles [Q1–Q2] vs. higher quartiles [Q3–Q4]) and hypertension status (absent vs. present), with the low-LAR/normotensive group serving as the reference. This analysis was used to estimate differences in cognitive outcomes between two exposure combinations, as well as to characterize the cognitive burden associated with their co-occurrence. Second, to evaluate multiplicative interaction, a product term (LAR_dichotomized × hypertension) was entered into the fully adjusted regression models together with the main effects. A *p*-value for interaction (*P*-interaction) < 0.05 was considered statistically significant. This analysis tested whether the association between LAR and cognitive outcomes differed by hypertension status on a multiplicative scale. These two approaches address distinct epidemiological questions: the joint exposure analysis evaluates outcome differences across exposure combinations, whereas the multiplicative interaction analysis tests for departure from multiplicativity. From a clinical perspective, the joint exposure analysis may help identify individuals with co-occurring elevated LAR and hypertension who have a higher cognitive burden, even if there is no statistically significant multiplicative interaction. For the clinical validation cohort, global cognitive function was also assessed using the Mini-Mental State Examination (MMSE) and the Montreal Cognitive Assessment (MoCA).

### Statistical analysis

Descriptive statistics are presented as weighted mean ± SD (continuous variables) or weighted percentages (categorical variables); between-group comparisons are via weighted *t*-test/ANOVA or Rao-Scott chi-square test, as appropriate. RCS regression was used to model non-linear relationships, with knots placed in the 5th, 50th, and 95th percentiles of the LAR distribution (Qiu et al., 2025). Multivariable linear regression models were adjusted incrementally: Model 1, unadjusted; Model 2, adjusted for age, race, education, marital status, alcohol consumption, smoking, and diabetes; Model 3, fully adjusted model, adjusted for all confounders of Model 2 and 12 laboratory parameters (e.g., glucose, creatinine, and lipid profile). Subgroup analyses were stratified by age (65–74 vs. ≥ 75 years), gender, diabetes status, drinking status, and smoking history. Kaplan–Meier curves with log-rank tests were used for survival analysis. Cox proportional hazards regression models were further constructed to evaluate the associations between exposure groups and survival outcomes, with effect estimates expressed as HRs and 95% CIs. The validation cohort was used by Mann–Whitney *U*-tests, multivariable logistic regression, and ROC analysis. All statistical analyses were performed using R version 4.4.2 (R Foundation for Statistical Computing, Vienna, Austria) and GraphPad Prism v10 (GraphPad Software, San Diego, CA, USA).

## Results

### Baseline characteristics

Weighted baseline characteristics of the NHANES cohort are summarized in Supplementary Table S1, revealing significant differences across groups in age, race, education, smoking, diabetes, hypertension, and laboratory tests [including low-density lipoprotein (LDL), leukocyte count, albumin, and triglycerides] (all *p* < 0.05). Participants in the elevated LAR + hypertension group were significantly older (70.12 ± 6.83 years), had higher white blood cell (WBC) counts (7.96 ± 1.52 × 10^9^/L), lower albumin levels (41.16 ± 2.85 g/L), and higher rates of diabetes (34.5%) and smoking (60.6%) compared to the normal group (all *p* < 0.001).

### LAR and cognitive function: non-linear and joint associations with hypertension

**Figure 2:**
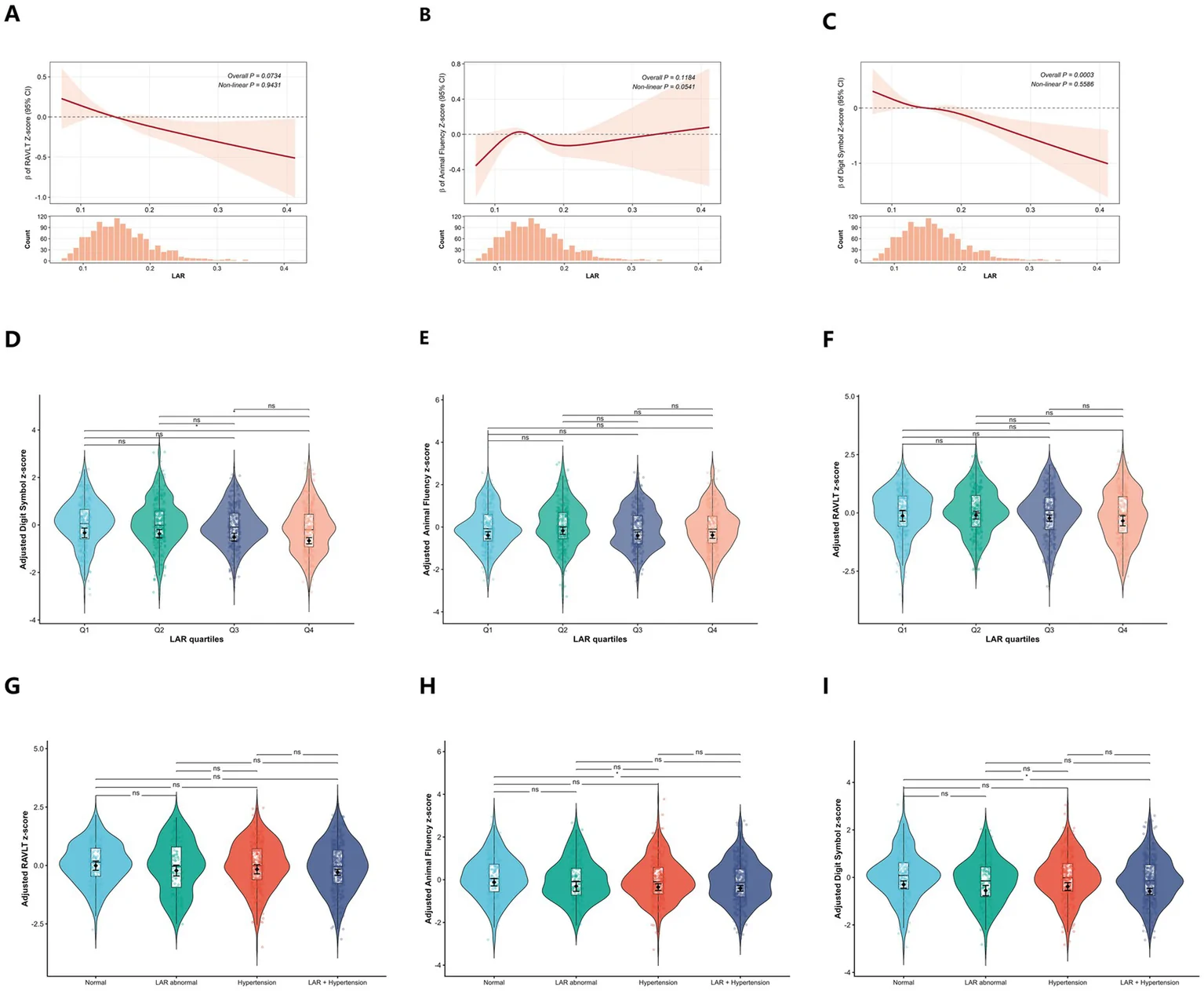
Relationships between leukocyte–albumin ratio (LAR) and cognitive function. **(A–C)** Restricted cubic spline (RCS) curves map LAR’s links with Rey Auditory Verbal Learning Test (RAVLT), animal fluency, and digit symbol substitution test (DSST) *z*-scores. **(D–F)** Violin plots display *z*-scores (adjusted for sex, age, race, education, marital status, alcohol use, smoking, diabetes) across LAR quartiles. **(G–I)** Violin plots display adjusted *z*-scores (RAVLT, animal fluency, DSST) combined across LAR-hypertension groups. *p < 0.05; ns, not significant.

RCS regression revealed no significant non-linear associations between LAR and cognitive *z*-scores for the RAVLT, animal fluency task, or DSST (non-linearity *p*-values: 0.889, 0.064, 0.645, respectively). While overall associations were non-significant for RAVLT (*p* = 0.072) and animal fluency (*p* = 0.136), a statistically significant linear relationship was observed for DSST (*p* < 0.001), supporting a primarily linear LAR-DSST correlation (Figures 2A–C). Consistently, multivariable linear regression stratified by LAR quartiles showed that, relative to Q1, Q4 was significantly linked to lower DSST performance in fully adjusted Model 3 (*β* = −0.318, 95% CI: −0.586 to −0.050, *p* = 0.027); no such associations were detected for RAVLT or animal fluency. Additionally, joint analysis of LAR status and hypertension demonstrated that the LAR + hypertension group was significantly associated with lower performance across all three cognitive domains in fully adjusted Model 3: RAVLT (*β* = −0.348, 95% CI: −0.627 to −0.070, *p* = 0.022), animal fluency (*β* = −0.330,95% CI: −0.565 to −0.096, *p* = 0.014), and DSST (*β* = −0.287, 95% CI: −0.509 to −0.066, *p* = 0.019) (Table 1, Supplementary Table S2).

**Table 1:** Association between LAR, hypertension, and cognitive function (NHANES cohort).

Variable	Model 1 *β* (95% CI), *p*	Model 2 *β* (95% CI), *p*	Model 3 *β* (95% CI), *p*
RAVLT			
LAR + hypertension	−0.360 (−0.573, −0.147), 0.002	−0.286 (−0.538, −0.035), 0.028	−0.348 (−0.627, −0.070), 0.022
Abnormal LAR	−0.245 (−0.484, −0.006), 0.045	−0.211 (−0.480, 0.059), 0.117	−0.231 (−0.519, 0.058), 0.098
Hypertension	−0.199 (−0.420, 0.022), 0.076	−0.155 (−0.388, 0.077), 0.175	−0.195 (−0.440, 0.050), 0.100
Animal fluency			
LAR + hypertension	−0.401 (−0.591, −0.210), <0.001	−0.289 (−0.485, −0.092), 0.007	−0.330 (−0.565, −0.096), 0.014
Abnormal LAR	−0.234 (−0.495, 0.026), 0.076	−0.189 (−0.424, 0.047), 0.110	−0.206 (−0.492, 0.079), 0.127
Hypertension	−0.318 (−0.539, −0.098), 0.006	−0.229 (−0.421, −0.037), 0.023	−0.268 (−0.509, −0.028), 0.034
DSST			
LAR + hypertension	−0.468 (−0.637, −0.298), <0.001	−0.290 (−0.480, −0.099), 0.005	−0.287 (−0.509, −0.066), 0.019
Abnormal LAR	−0.331 (−0.594, −0.068), 0.015	−0.252 (−0.490, −0.014), 0.039	−0.235 (−0.525, 0.055), 0.095
Hypertension	−0.221 (−0.428, −0.014), 0.037	−0.082 (−0.260, 0.095), 0.339	−0.094 (−0.282, 0.095), 0.271

Model 1: unadjusted. Model 2: adjusted for age, sex, race, education level, marital status, alcohol consumption, smoking, and diabetes. Model 3: adjusted for all confounders of Model 2 and 12 lab data (red blood cells, hemoglobin, platelets, alkaline phosphatase, total calcium, creatinine, alanine aminotransferase, iron, total bilirubin, and uric acid). RAVLT, Rey Auditory Verbal Learning Test; LAR, leukocyte–albumin ratio; DSST, digit symbol substitution test; CI, confidence interval.

Formal interaction analysis showed that the multiplicative interaction term (LAR_dichotomized × hypertension) was not statistically significant for any of the three cognitive domains (all *P* for interaction > 0.05), indicating no evidence of multiplicative interaction. However, in the joint exposure analysis, participants with increased LAR and hypertension had significantly lower scores than the reference group in all three cognitive domains, indicating a clinically relevant joint association. (Supplementary Table S3).

### Cognitive *Z*-score distributions by LAR quartiles and combined LAR-hypertension status

Violin plots visualized the distribution of cognitive *z*-scores—adjusted for sex, age, race/ethnicity, education, marital status, alcohol use, smoking status, and diabetes—across LAR quartiles and combined LAR-hypertension groups (Figures 2D–I). Across LAR quartiles, no significant differences in RAVLT or animal fluency *z*-scores were observed between groups. In contrast, DSST *z*-scores were significantly lower in the highest LAR quartile (Q4) relative to the lowest (Q1) (*p* < 0.05), indicating that elevated LAR levels are correlated with reduced processing speed. In the combined LAR-hypertension analysis, DSST *z*-scores were significantly lower in the abnormal LAR group vs. the normal LAR group (*p* < 0.05). No significant group differences were detected in RAVLT or animal fluency *z*-scores across any comparison. These results demonstrate that the association between LAR and cognitive function is most evident for processing speed (assessed via DSST) and is detectable independent of hypertension status.

### Subgroup analysis

**Figure 3:**
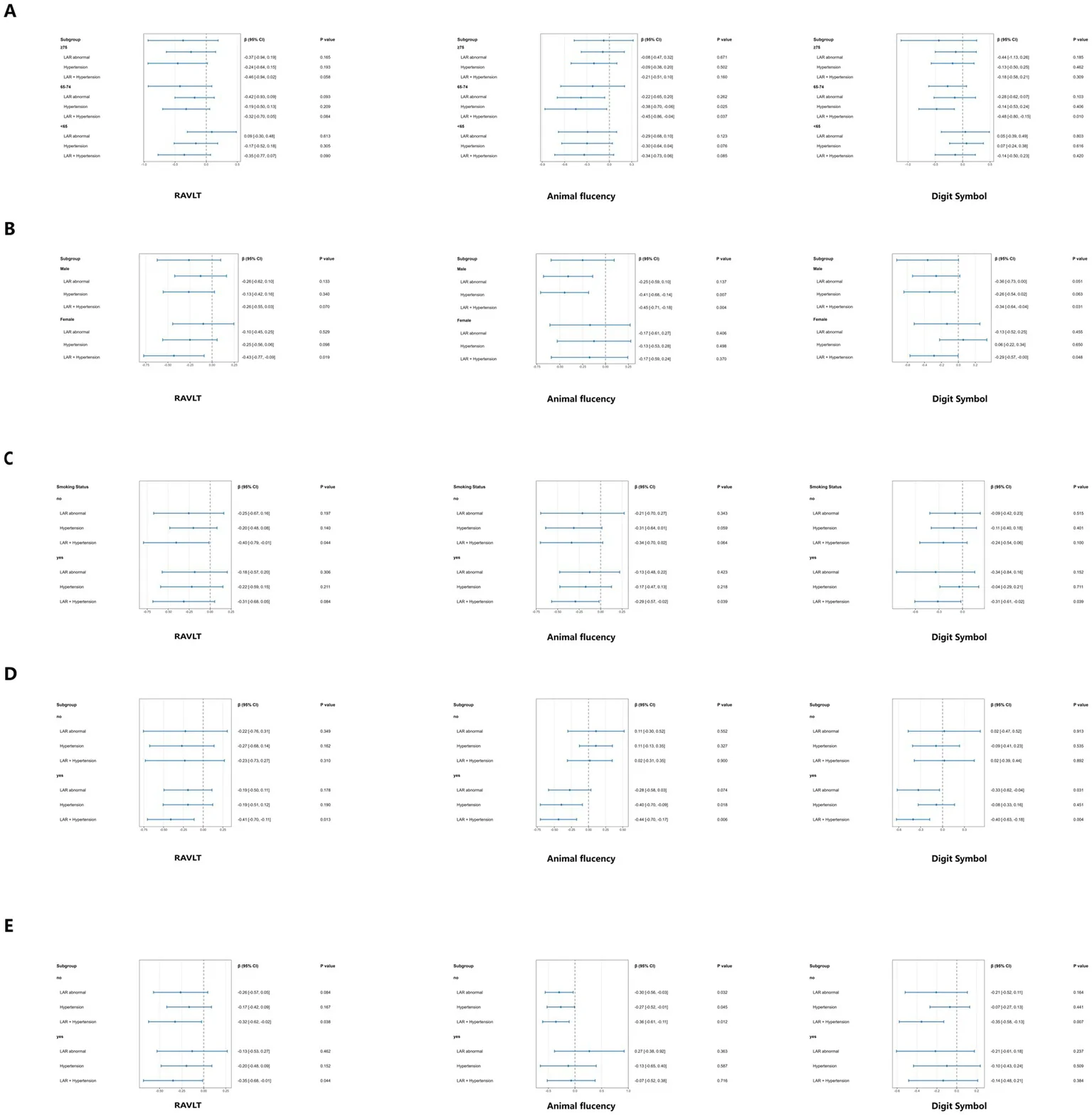
Forest plots of subgroup analyses for LAR/hypertension status and cognitive performance. Subgroup analyses stratified by **(A)** age, **(B)** sex, **(C)** smoking status, **(D)** alcohol consumption, and **(E)** diabetes status evaluated associations of leukocyte–albumin ratio (LAR) abnormality, hypertension, and their combinations (LAR + hypertension) with cognitive performance across three domains: Rey Auditory Verbal Learning Test (RAVLT), animal fluency, and digit symbol substitution test (DSST). Effect sizes are reported as *β* coefficients with 95% confidence intervals (CIs).

Subgroup analyses examined whether combined LAR abnormality and hypertension were differentially associated with cognitive function across age, sex, smoking, alcohol use, and diabetes status (Figures 3A–E). In adults aged 65–74 years, LAR + hypertension was significantly correlated with lower animal fluency (*β* = −0.45, 95% CI: −0.86 to −0.04, *p* = 0.037) and DSST scores (*β* = −0.48, 95% CI: −0.80 to −0.15, *p* = 0.010), with no effects in < 65 or ≥ 75-year-olds. Sex-specific patterns emerged: males showed associations between LAR + hypertension (*β* = −0.45, *p* = 0.004) or isolated hypertension (*β* = −0.41, *p* = 0.007) and poorer animal fluency; females exhibited associations with reduced RAVLT (*β* = −0.43, *p* = 0.019) and DSST scores (*β* = −0.29, *p* = 0.048). Among current/former smokers, LAR + hypertension was associated with lower DSST (*β* = −0.31, *p* = 0.039) and animal fluency (*β* = −0.26, *p* = 0.009); drinkers showed deficits in RAVLT (*β* = −0.41, *p* = 0.013), animal fluency (*β* = −0.44, *p* = 0.006), and DSST (*β* = −0.40, *p* = 0.004). Non-diabetic individuals had significant impairments in all three cognitive domains in association with LAR + hypertension, while no associations appeared in those with diabetes. Overall, adverse cognitive associations were most pronounced in males (semantic memory), females (memory/processing speed), smokers, drinkers, and non-diabetic individuals, suggesting effect modification by these factors.

### Survival analysis

**Figure 4:**
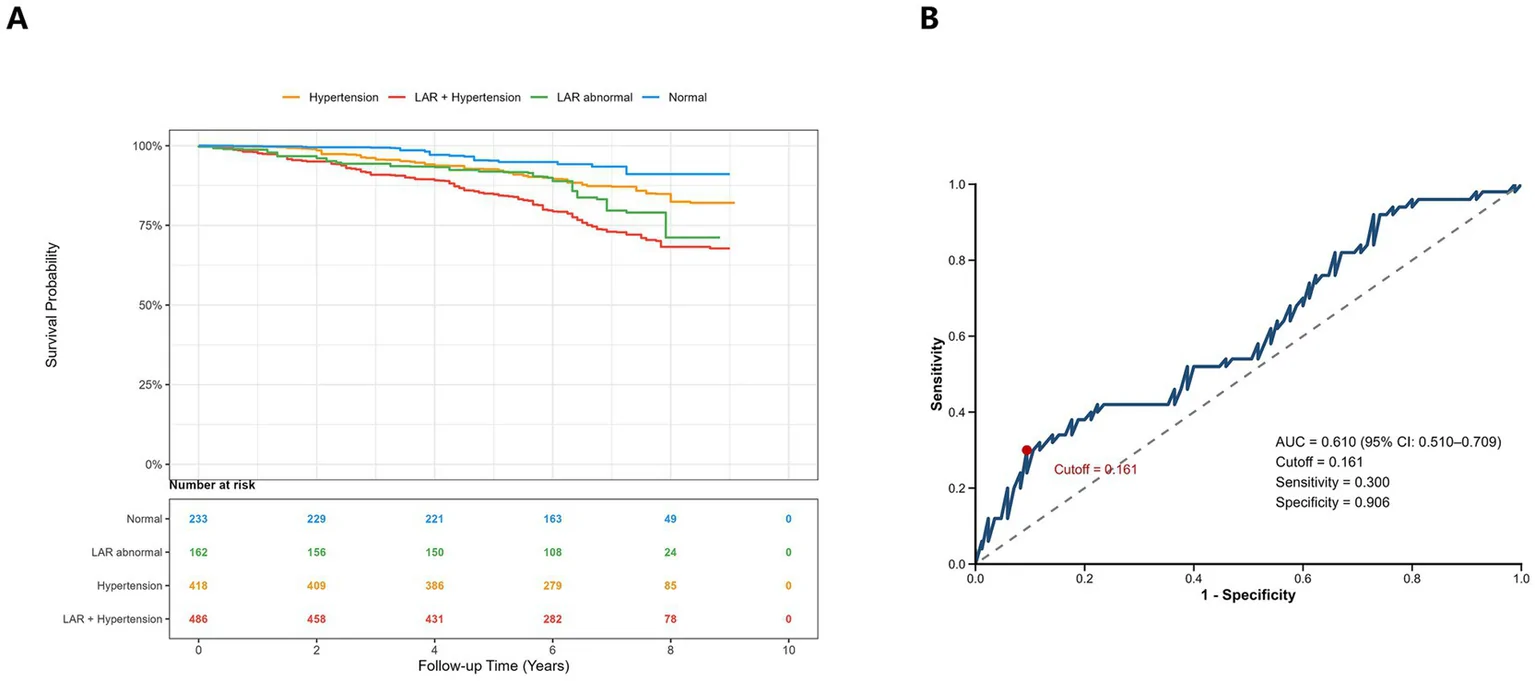
Kaplan–Meier curves for all-cause mortality and receiver operating characteristic (ROC) curve. **(A)** Kaplan–Meier curves for all-cause mortality stratified by LAR abnormality and hypertension status. **(B)** ROC curve of the predictive model in the validation cohort. During follow-up, the normal group (no LAR abnormality or hypertension) showed the highest survival probability, while the group with concurrent LAR abnormality and hypertension had the lowest. Numbers at risk are displayed beneath the curves. The area under the curve (AUC) with 95% confidence interval (CI) is shown, together with the optimal cutoff value determined by Youden’s index.

Kaplan–Meier survival curves revealed distinct separation among four groups during follow-up (Figure 4A). The normotensive group with normal LAR had the highest survival probability, while participants with both LAR abnormality and hypertension exhibited the lowest. Isolated LAR abnormality or hypertension was correlated with intermediate outcomes, with the co-exposure group showing the poorest survival trajectory. In unadjusted Cox models, isolated LAR abnormality (HR = 2.67, 95% CI: 1.25–5.71, *p* = 0.011) and the combined group (HR = 4.02, 95% CI: 2.15–7.51, *p* < 0.001) had significantly elevated all-cause mortality risk vs. controls; isolated hypertension was non-significant (HR = 1.91, 95% CI: 0.97–3.77, *p* = 0.061). After partial adjustment, only the co-exposure group remained significant (HR = 2.52, 95% CI: 1.29–4.94, *p* = 0.007). In fully adjusted models, the combined group still had a significant association with mortality risk (HR = 2.17, 95% CI: 1.12–4.18, *p* = 0.021), while isolated LAR abnormality was borderline (HR = 2.11, 95% CI: 0.96–4.62, *p* = 0.063), and hypertension remained non-significant (HR = 1.18, 95% CI: 0.63–2.22, *p* = 0.611) (Supplementary Table S4). Overall, the co-occurrence of LAR abnormality and hypertension was independently associated with the highest all-cause mortality risk.

### Validation cohort: LAR and AD risk

AD patients had significantly higher median LAR levels than age- and gender-matched controls (0.138 vs. 0.131, *p* = 0.033; Table 2). Multivariable logistic regression revealed the highest LAR quartile (Q4) was associated with 3.92-fold increased odds of AD (OR = 3.921, 95% CI: 1.336–12.361, *p* = 0.015; Table 3). ROC curve analysis demonstrated an area under the curve (AUC) of 0.61 (95% CI: 0.51–0.71), indicating moderate discriminative ability, with an optimal cutoff of 0.161 (sensitivity = 30.0%, specificity = 90.6%; Figure 4B). Intra-AD subgroup analyses showed no significant differences in global cognitive scores, including the MMSE and MoCA (Supplementary Table S5), likely due to the small sample size.

**Table 2:** Comparison of LAR between healthy groups and the elderly AD group.

Group	Index_value	*Z*-value	*p*-value
Healthy group (< 60y)	0.121 (0.101, 0.144)	−0.746	0.456^#^
Healthy group (≥ 60y)	0.131 (0.1048, 0.145)	−2.128	0.033^*^
AD group (≥ 60y)	0.138 (0.114, 0.163)	−2.128	0.033^*^

Wilcoxon rank-sum test was used; AD, Alzheimer’s disease; ^#^comparison result with the healthy group (≥ 60 years); *comparison result with the AD group.

**Table 3:** AD risk by LAR quartiles (validation cohort).

Variable	OR (95% CI)	*p*-value
LAR quartile (Q1, reference)	1	/
Q2 (low-moderate LAR)	1.771 (0.605–5.369)	0.301
Q3 (moderate-high LAR)	0.928 (0.292–2.931)	0.897
Q4 (high LAR)	3.921 (1.336–12.361)	0.015^*^
Gender (female, reference)		
Male	0.614 (0.274–1.335)	0.225
Age (per 1-year increase)	1.129 (1.057–1.212)	<0.001^***^

*p < 0.05, ***p < 0.001. OR, odds ratio; CI, confidence interval. Adjusted for gender and age.

## Discussion

To our knowledge, this is the first study to show that the co-occurrence of elevated LAR and hypertension was associated with poor multidimensional cognitive performance and higher AD risk in older adults. These findings contribute to AD pathogenesis research by highlighting the co-occurring and additive roles of vascular dysfunction and systemic inflammation-modifiable early factors in neurodegeneration (Pilotto et al., 2021; Mayer and Fischer, 2025).

Mechanistically, hypertension disrupts blood–brain barrier (BBB) integrity, enabling inflammatory cell infiltration, while hypoalbuminemia impairs anti-inflammatory defenses and neuronal repair (Kluger et al., 2023). This pro-inflammatory, nutritionally compromised state may amplify vascular damage and cognitive decline (Severinsen and Jakobsen, 2007; Woeffler-Maucler et al., 2014), aligning with AD pathophysiology models emphasizing vascular-neuroinflammatory crosstalk (Miao et al., 2025). Our results support a framework in which hypertension and elevated LAR may jointly contribute to cognitive decline (the joint presence of both risk factors was associated with the greatest cognitive burden, which is consistent with an additive risk model), though causal relationships cannot be established from the present data. Limitations include potential overestimation of synergistic associations due to unaccounted healthy lifestyle confounders and lack of hypertension severity data, warranting future exploration.

Subgroup analyses identified two high-susceptibility populations: adults aged 65–74 years, in whom vascular-inflammatory risks may exert peak impact on cognitive decline during the preclinical AD stage (Smith et al., 2025), and females, whose post-menopausal estrogen deficiency increases BBB permeability by 30–40% (Rettberg et al., 2014), potentially amplifying the effects of hypertension and systemic inflammation on brain function and reducing anti-inflammatory responses (Carroll et al., 2016). Even after accounting for increased life expectancy, females have higher rates of AD than males, and recent studies have shown that systemic inflammatory markers have stronger associations with cognitive impairment in females (Ng Wing Tin et al., 2019). These observations support age- and sex-tailored prevention strategies, pending replication in independent cohorts (Proietti et al., 2025).

Clinically, LAR may provide a more integrated reflection of systemic inflammation and nutritional status than either marker alone (Lowry et al., 2022). In our study, its association with cognitive performance appeared predominantly linear, particularly for DSST. This threshold effect is consistent with composite biomarker studies in AD, where combined markers often exhibit non-linear associations with disease progression (Xu et al., 2022). In the validation cohort, LAR demonstrated a statistically significant association with AD risk (Q4 vs. Q1: OR = 3.92, *p* = 0.015); however, its discriminative performance was modest, with an area under the ROC curve of 0.61 (95% CI: 0.51–0.71). This AUC indicates that LAR alone has limited ability to distinguish AD patients from cognitively healthy controls, correctly classifying only 61% of pairs. At the optimal cutoff of 0.161, sensitivity was low (30.0%), though specificity was high (90.6%). These results suggest that LAR is unlikely to serve as a standalone diagnostic biomarker. Rather, its potential clinical value may lie in its high specificity, which could be useful as a preliminary screening tool in resource-limited primary care settings to identify individuals warranting further evaluation or as one component of a multi-marker risk stratification panel. We caution against overinterpreting these results and emphasize that LAR’s modest discriminative ability precludes its use as a replacement for established AD biomarkers (e.g., CSF Aβ/p-tau, amyloid PET). Future studies incorporating LAR alongside other low-cost biomarkers may help determine whether a composite panel can achieve clinically meaningful discriminative performance.

Biologically, LAR may drive AD through several potential pathways: impaired Aβ clearance (hypoalbuminemia reduces BBB transport), tau hyperphosphorylation (inflammation activates glial cells), and exacerbated white matter injury (hypertension + inflammation) (Boada et al., 2020; Jacobs et al., 2024; Low et al., 2019). Our study conjectures that elevated LAR (reflecting systemic inflammation and malnutrition) combined with hypertension may contribute to AD via synergistic inflammatory-vascular mechanisms. However, the cross-sectional design limits causal inference; future longitudinal studies with AD biomarkers (e.g., amyloid PET, CSF tau) are needed to validate these associations.

Our study’s robustness and translational relevance are supported by three key features: (1) Dual-cohort design: A nationally representative NHANES cohort (n = 1,300) ensures generalizability to community-dwelling older adults, while a real-world clinical validation cohort confirms LAR’s utility as an AD risk stratification biomarker. (2) Multidimensional cognitive assessment: Domain-specific tests and the MMSE detect subtle preclinical deficits before overt dementia onset (Jaeger, 2018). Multiplicative interaction terms in fully adjusted models were used to evaluate synergistic associations between LAR and hypertension, while minimizing residual bias (Knol and Vanderweele, 2012). (3) Clinical utility: LAR is a low-cost, widely available plasma biomarker derived from routine labs, suitable for large-scale AD risk stratification in primary care and resource-limited settings, though its modest discriminative ability (AUC = 0.61) should be recognized.

Several limitations warrant consideration. The cross-sectional design cannot establish causality; longitudinal studies are needed to confirm temporality. The small validation cohort limits subgroup analyses; generalizability is limited to northern Han Chinese populations. Beyond the cross-sectional design and small validation cohort, three additional limitations warrant consideration. First, residual confounding remains possible. We lack data on key variables, including the APOE ε4 genotype, specific medication use (e.g., statins, antihypertensives), lifestyle factors (e.g., diet, exercise), and socioeconomic status, all of which may modulate LAR and cognitive outcomes. Second, we cannot rule out reverse causation. Preclinical AD pathology might itself induce systemic inflammation and nutritional changes, making elevated LAR a consequence rather than a cause, highlighting the need for longitudinal validations. Third, unmeasured frailty and comorbidity burdens—inherent to age-related physiological decline—may confound the observed associations. Future research incorporating comprehensive geriatric assessments is essential to determine whether LAR and hypertension independently affect cognition beyond general health status. Additionally, missing AD biomarkers (Aβ, tau, neuroimaging), unmeasured confounders (lifestyle, socioeconomic variables), and interventional data (medication use) restrict mechanistic insights. Our findings on LAR’s association with AD risk are preliminary. Validation in large multi-center prospective cohorts, paired with comprehensive data, stratification, and *in vivo* AD biomarkers (CSF Aβ/p-tau), is critical to clarify underlying pathological mechanisms and evaluate targeted interventions.

## Conclusion

Our findings support an association between LAR, hypertension, and cognitive decline. Elevated LAR, particularly in combination with hypertension, is associated with poor cognitive performance across multiple domains in older adults, as observed in both the NHANES and a clinical AD cohort. As a low-cost, easily measurable potential biomarker, LAR shows modest discriminative ability for AD risk stratification (AUC = 0.61); however, its limited sensitivity (30.0%) indicates that it should not be used in isolation but rather as a component of broader risk assessment, particularly in primary care settings where advanced biomarkers are unavailable. Prospective longitudinal studies with larger sample sizes are urgently needed to validate the relationships of LAR with cognitive decline and to translate these findings into clinical practice.

## Data Availability

The NHANES datasets analyzed in this study are publicly available in the CDC repository at https://www.cdc.gov/nchs/nhanes/index.html. The validation cohort datasets used and/or analyzed during the current study are available from the corresponding author (JC, 13683581168@126.com) upon reasonable request and after institutional review board approval.

## Footnotes

1
https://www.cdc.gov/nchs/nhanes/about/erb.html?CDC_AAref_Val=https://www.cdc.gov/nchs/nhanes/irba98.htm
