# Supplementary material for: Joint effects of leukocyte-albumin ratio and hypertension are associated with multidimensional cognitive decline and Alzheimer’s disease risk in older adults: evidence from NHANES and a clinical AD validation cohort
Source: Front Aging Neurosci. 2026 Aug 12;18:1826100. doi: 10.3389/fnagi.2026.1826100 (PMC13506719; doi:10.3389/fnagi.2026.1826100)
Supplement: Supplementary file 1 [file Supplementary_file_1.docx]

**Supplementary Table S1. Weighted Baseline Characteristics of Participants Stratified by LAR and Hypertension Status**

| Variable | Normal | Elevated LAR | Hypertension | Elevated LAR + Hypertension | *P* value |
| --- | --- | --- | --- | --- | --- |
|  | *n=233* | *n=163* | *n=418* | *n=486* |  |
| Age（years） | 67.13 （5.53） | 69.15 （6.24） | 69.58 （6.71） | 70.12 （6.83） | <0.001 |
| age，n（%） |  |  |  |  | 0.012 |
| 65-74 | 106 （48.7%） | 63 （48.9%） | 176 （39.9%） | 201 （41.5%） |  |
| <65 | 87 （38.4%） | 55 （27.8%） | 125 （32.3%） | 127 （28.8%） |  |
| ≥75 | 40 （12.9%） | 45 （23.3%） | 117 （27.8%） | 158 （29.7%） |  |
| Sex，n（%） |  |  |  |  | 0.161 |
| Male | 130 （54.5%） | 101 （60.4%） | 216 （54.9%） | 264 （58.5%） |  |
| Female | 103 （45.5%） | 62 （39.6%） | 202 （45.1%） | 222 （41.5%） |  |
| Race，n（%） |  |  |  |  | <0.001 |
| Mexican American | 20 （2.4%） | 22 （4.5%） | 23 （2.2%） | 50 （4.2%） |  |
| Non-Hispanic Black | 28 （2.8%） | 11 （1.8%） | 120 （10.1%） | 102 （8.2%） |  |
| Non-Hispanic White | 131 （88.2%） | 90 （85.7%） | 194 （79.2%） | 250 （77.9%） |  |
| Other Hispanic | 26 （3.0%） | 28 （5.1%） | 37 （3.4%） | 45 （3.4%） |  |
| Other Race - Including Multi-Racial | 28 （3.6%） | 12 （3.0%） | 44 （5.2%） | 39 （6.4%） |  |
| Education，n（%） |  |  |  |  | <0.001 |
| University and above | 144 （75.0%） | 76 （55.0%） | 229 （63.7%） | 212 （53.6%） |  |
| High school | 43 （17.8%） | 39 （22.8%） | 93 （21.6%） | 133 （26.1%） |  |
| <High school | 46 （7.2%） | 48 （22.2%） | 96 （14.7%） | 141 （20.3%） |  |
| Marital status，n（%） |  |  |  |  | 0.047 |
| Unmarried/Widowed/Divorced/ Separated | 75 （22.9%） | 56 （31.6%） | 156 （30.7%） | 205 （37.3%） |  |
| Married/living with partner | 158 （77.1%） | 107 （68.4%） | 262 （69.3%） | 281 （62.7%） |  |
| Alcohol users，n（%） |  |  |  |  | 0.839 |
| No | 71 （23.6%） | 54 （34.6%） | 142 （28.9%） | 160 （28.9%） |  |
| Yes | 162 （76.4%） | 109 （65.4%） | 276 （71.1%） | 326 （71.1%） |  |
| Smoking，n（%） |  |  |  |  | <0.001 |
| No | 139 （57.4%） | 68 （48.4%） | 229 （52.8%） | 207 （39.4%） |  |
| Yes | 94 （42.6%） | 95 （51.6%） | 189 （47.2%） | 279 （60.6%） |  |
| Diabetes，n（%） |  |  |  |  | <0.001 |
| No | 206 （92.2%） | 118 （78%） | 292 （74.5%） | 290 （65.5%） |  |
| Yes | 27 （7.8%） | 45 （22.0%） | 126 （25.5%） | 196 （34.5%） |  |
| TG（mmol/L） | 1.10 （0.52） | 1.30 （0.60） | 1.34 （0.70） | 1.59 （0.82） | <0.001 |
| LDL（mmol/L） | 3.15 （0.82） | 2.88 （0.88） | 2.86 （0.95） | 2.72 （0.94） | 0.001 |
| HDL（mmol/L） | 1.63 （0.41） | 1.45 （0.40） | 1.51 （0.47） | 1.36 （0.40） | <0.001 |
| TC（mmol/L） | 5.28 （0.94） | 4.93 （1.00） | 4.99 （1.14） | 4.81 （1.04） | <0.001 |
| WBC（×10^9^/L） | 5.06 （0.90） | 7.92 （1.45） | 5.35 （0.85） | 7.96 （1.52） | <0.001 |
| LY（×10^9^/L） | 1.54 （0.41） | 2.10 （0.79） | 1.57 （0.43） | 1.98 （0.67） | <0.001 |
| MONO（×10^9^/L） | 0.46 （0.12） | 0.64 （0.17） | 0.50 （0.38） | 0.62 （0.19） | <0.001 |
| NEUT（×10^9^/L） | 2.85 （0.78） | 4.77 （1.20） | 3.06 （0.77） | 5.07 （1.45） | <0.001 |
| RBC（×10^12^/L） | 4.49 （0.42） | 4.68 （0.45） | 4.47 （0.46） | 4.53 （0.49） | 0.002 |
| HGB（g/dL） | 14.13 （1.18） | 14.58 （1.40） | 13.95 （1.37） | 14.01 （1.46） | 0.001 |
| PLT（×10^9^/L） | 206.01 （42.98） | 236.15 （58.54） | 214.01 （52.87） | 232.29 （66.29） | <0.001 |
| GLU（mmol/L） | 5.70 （1.50） | 6.24 （1.61） | 6.24 （1.68） | 6.62 （1.95） | 0.002 |
| HbA1c（%） | 5.59 （0.48） | 6.05 （1.20） | 5.91 （0.89） | 6.16 （1.10） | <0.001 |
| ALT（U/L） | 22.49 （14.28） | 20.55 （8.48） | 23.14 （13.33） | 22.01 （10.58） | 0.14 |
| AST（U/L） | 24.70 （7.95） | 23.61 （7.75） | 26.10 （11.50） | 24.58 （9.82） | 0.166 |
| ALP（U/L） | 61.66 （16.58） | 65.85 （21.38） | 66.68 （22.49） | 70.83 （22.12） | 0.007 |
| BUN（mmol/L） | 5.24 （1.60） | 5.32 （2.14） | 5.72 （2.14） | 6.23 （2.98） | <0.001 |
| Ca^2+^（mmol/L） | 2.35 （0.08） | 2.34 （0.07） | 2.36 （0.13） | 2.35 （0.11） | 0.138 |
| CK（IU/L） | 118.14 （68.41） | 114.56 （87.60） | 120.93 （92.51） | 105.35 （75.97） | 0.257 |
| CR（umol/L） | 76.92 （15.93） | 83.49 （36.49） | 87.79 （59.03） | 95.46 （89.31） | <0.001 |
| γGGT（U/L） | 18.21 （10.35） | 23.21 （21.20） | 26.95 （27.83） | 30.64 （38.66） | <0.001 |
| Fe（umol/L） | 17.88 （5.72） | 15.65 （5.16） | 16.46 （5.84） | 15.32 （5.19） | <0.001 |
| LDH（U/L） | 128.96 （21.36） | 133.46 （55.19） | 132.66 （25.10） | 134.15 （29.57） | 0.121 |
| P（mmol/L） | 1.17 （0.19） | 1.19 （0.18） | 1.19 （0.17） | 1.22 （0.22） | 0.162 |
| TBIL（umol/L） | 12.96 （4.24） | 12.50 （4.19） | 12.77 （4.77） | 11.31 （4.02） | <0.001 |
| TP（mean（SD）） | 69.13 （3.75） | 69.96 （4.78） | 69.51 （4.54） | 69.83 （4.64） | 0.179 |
| UA（mean（SD）） | 301.91 （69.53） | 318.80 （71.09） | 337.76 （80.80） | 360.31 （93.75） | <0.001 |
| Na^+^（mmol/L） | 140.01 （1.99） | 139.73 （2.49） | 139.75 （2.61） | 139.56 （2.73） | 0.222 |
| CL^-^（mmol/L） | 104.47 （2.41） | 103.92 （3.00） | 103.77 （3.14） | 103.52 （3.30） | 0.006 |
| Globin（g/L） | 25.98 （3.62） | 28.88 （4.76） | 26.94 （4.38） | 28.67 （4.54） | <0.001 |
| APOB（mg/dL） | 0.92 （0.21） | 0.90 （0.23） | 0.88 （0.23） | 0.90 （0.24） | 0.673 |
| ALB（g/L） | 43.15 （2.20） | 41.08 （2.82） | 42.57 （2.78） | 41.16 （2.85） | <0.001 |

Note: Elevated LAR defined as > 0.142 (75^th^ percentile of the study population).

Abbreviations: LAR, Leukocyte-to-Albumin Ratio; TG: Triglyceride; LDL: Low-density lipoprotein; HDL: High-density lipoprotein; TC: Total cholesterol; WBC: White blood cell; LY: Lymphocyte; MONO: Monocyte; NEUT: Neutrophil; RBC: Red blood cell; HGB: Hemoglobin; PLT: Platelet; GLU: Glucose; HbA1c: Glycated hemoglobin; BUN: Blood urea nitrogen; Ca: Calcium ion; CK: Creatine kinase; CR: Creatinine; γGGT: γ-Glutamyl transferase; Fe: Iron ion; LDH: Lactate dehydrogenase; P: Phosphorus; UA: Uric acid; Na: Sodium ion; K: Potassium ion; CL: Chloride ion; ALT: Alanine aminotransferase; AST: Aspartate aminotransferase; ALP: Alkaline phosphatase; APOB: Apolipoprotein B; ALB: Albumin.

**Supplementary Table S2. Multivariable Linear Regression Analysis of the Association Between LAR Quartiles and Cognitive Function**

| Variable | Model 1 β (95% CI), *P* | Model 2 β (95% CI), *P* | Model 3 β (95% CI), *P* |
| --- | --- | --- | --- |
| **RAVLT** |  |  |  |
| Q2 | 0.004 (−0.217, 0.225), 0.969 | 0.026 (−0.215, 0.268), 0.820 | −0.015 (−0.253, 0.224), 0.886 |
| Q3 | −0.151 (−0.392, 0.090), 0.210 | -0.103 (-0.374, 0.167), 0.430 | −0.151 (−0.423, 0.121), 0.224 |
| Q4 | −0.279 (−0.527, -0.032), 0.028 | −0.220 (−0.539, 0.100), 0.164 | -0.263 (-0.598, 0.072), 0.103 |
| **animal fluency** |  |  |  |
| Q2 | 0.210 (0.005, 0.414), 0.045 | 0.217 (0.008, 0.425), 0.042 | 0.203 (−0.040, 0.446), 0.087 |
| Q3 | −0.062 (−0.292, 0.168), 0.586 | −0.011 (−0.249, 0.227), 0.924 | −0.031 (−0.293, 0.232), 0.786 |
| Q4 | −0.065 (−0.236, 0.106), 0.442 | 0.006 (−0.193, 0.206), 0.947 | 0.035 (−0.187, 0.257), 0.714 |
| **DSST** |  |  |  |
| Q2 | 0.210 (0.005, 0.414), 0.045 | −0.039 (−0.305, 0.227), 0.760 | −0.044 (−0.318, 0.230), 0.706 |
| Q3 | −0.062 (−0.292, 0.168), 0.586 | −0.183 (−0.430, 0.064), 0.137 | −0.181 (−0.449, 0.086), 0.148 |
| Q4 | −0.065 (−0.236, 0.106), 0.442 | −0.332 (−0.560, -0.104), 0.007 | −0.318 (−0.586, -0.050), 0.027 |

Model 1: Unadjusted. Model 2: Adjusted for age, sex, race, education level, marital status, alcohol consumption, smoking, and diabetes. Model 3: Adjusted for all confounders of Model 2 and 12 lab data (red blood cells, hemoglobin, platelets, alkaline phosphatase, total calcium, creatinine, alanine aminotransferase, iron, total bilirubin, and uric acid).

Abbreviations: LAR, Leukocyte-to-Albumin Ratio; DSST, Digit Symbol Substitution Test; *CI*, confidence interval.

Supplementary Table S3. Multiplicative Interaction Between LAR Status and Hypertension on Cognitive Domain Z-Scores (Fully Adjusted Model)

| Cognitive Domain | Interaction Term | Estimate | Std. Error | *t* value | *P* value |
| --- | --- | --- | --- | --- | --- |
| RAVLT | LAR (High) × Hypertension | 0.0773 | 0.1397 | 0.5529 | 0.6003 |
| Animal Fluency | LAR (High) × Hypertension | 0.1446 | 0.1368 | 1.0564 | 0.3315 |
| DSST | LAR (High) × Hypertension | 0.0411 | 0.1259 | 0.3267 | 0.7550 |

Abbreviations: LAR, leukocyte-albumin ratio; RAVLT, Rey Auditory Verbal Learning Test; DSST, Digit Symbol Substitution Test. Adjusted for age, sex, race, education level, marital status, alcohol consumption, smoking, diabetes, and 12 laboratory parameters. *P*-interaction > 0.05 for all domains.

**Supplementary Table S4. Cox Proportional Hazards Analysis of LAR Status and Hypertension on All-Cause Mortality**

| Variable | Model 1 HR (95% CI), *P* | Model 2 HR (95% CI), *P* | Model 3 HR (95% CI), *P* |
| --- | --- | --- | --- |
| Elevated LAR | 2.674 (1.252, 5.712), 0.011 | 1.942 (0.849, 4.443), 0.116 | 2.108 (0.961, 4.624), 0.063 |
| Hypertension | 1.913 (0.972, 3.767), 0.061 | 1.294 (0.663, 2.526), 0.450 | 1.178 (0.626, 2.216), 0.611 |
| Elevated LAR + Hypertension | 4.019 (2.150, 7.512), <0.001 | 2.522 (1.287, 4.941), 0.007 | 2.167 (1.123, 4.181), 0.021 |

Model 1: Unadjusted. Model 2: Adjusted for age, sex, race, education level, marital status, alcohol consumption, smoking, and diabetes. Model 3: Adjusted for all confounders of Model 2 and 12 lab data (red blood cells, hemoglobin, platelets, alkaline phosphatase, total calcium, creatinine, alanine aminotransferase, iron, total bilirubin, and uric acid).

Abbreviations: LAR, Leukocyte-to-Albumin Ratio; HR, Hazard Ratio.

**Supplementary Table S5. Intra-AD Cohort Cognitive Score Comparisons**

| Variables | Normal (n = 18) | High LAR (n = 13) | Hypertension (n = 7) | Abnormal (n = 12) | *F* | *P* |
| --- | --- | --- | --- | --- | --- | --- |
| MMSE score | 18.67 ± 7.05 | 18.62 ± 6.85 | 16.71 ± 6.99 | 18.08 ± 7.00 | 0.149 | 0.930 |
| MoCA score | 15.39 ± 6.71 | 14.69 ± 6.10 | 13.29 ± 7.36 | 13.75 ± 8.11 | 0.215 | 0.885 |

Note: AD participants were stratified into four groups: Normal (low LAR [Q1+Q2], no hypertension), Isolated High LAR (high LAR [Q3+Q4], no hypertension), Hypertension (low LAR [Q1+Q2], with hypertension), and Combined Abnormal (high LAR [Q3+Q4], with hypertension). No significant differences were observed in MMSE and MoCA scores among these groups (*P* > 0.05). Data are expressed as mean ± SD.

Abbreviations: AD, Alzheimer's Disease; LAR, Leukocyte-to-Albumin Ratio; MMSE, Mini-Mental State Examination; MoCA, Montreal Cognitive Assessment.
